# A Method for Detecting Preliminary Actions During an Actual Karate Kumite Match

**DOI:** 10.3390/s25134134

**Published:** 2025-07-02

**Authors:** Kwangyun Kim, Shuhei Tsuchida, Tsutomu Terada, Masahiko Tsukamoto

**Affiliations:** 1 Graduate School of Engineering, Kobe University, 1-1 Rokkodai-Cho, Nada-Ku, Kobe 657-8501, Hyogo, Japan; kwangyun-kim@stu.kobe-u.ac.jp (K.K.); tuka@kobe-u.ac.jp (M.T.); 2 Center for Interdisciplinary AI and Data Science, Ochanomizu University, 2-1-1 Otsuka, Bunkyo-Ku, Tokyo 112-8610, Japan; tsuchida.shuhei@ocha.ac.jp

**Keywords:** human motion analysis, karate, inertial sensor, sports support, autocorrelation function, dynamic time warping

## Abstract

Kumite is a karate sparring competition in which two players fight each other using various techniques. In kumite matches, it is essential to reduce a preliminary action (hereinafter referred to as “pre-action”), such as pulling the arms and lowering the shoulders just before performing an attack technique. This is because pre-actions reveal the timing of the attack to the opponent. However, players often find it difficult to recognize their own pre-actions, and accurately estimating their presence or absence is challenging with conventional motion analysis methods, as pre-actions are subtle compared to major techniques like punching or kicking. Previously, we proposed a method for detecting pre-actions during single punches performed in a static state using inertial sensors. While this method was effective in controlled situations, it failed to detect pre-actions in punches during actual kumite matches. The main reason is that players generally perform footwork during matches, and this footwork is often misrecognized as pre-action via conventional detection methods. To address misrecognition caused by footwork, we propose a new method that combines preprocessing designed to detect and smooth footwork segments in the inertial data with the conventional pre-action detection method, thereby enabling pre-action detection during kumite matches. In the preprocessing, we apply an autocorrelation function to assess the constancy of footwork and accurately separate the footwork segment from the kumite technique segment. Only the footwork segment is then smoothed to suppress its influence on the detection process. Our experimental results show that the proposed method can estimate the presence or absence of pre-action in the punch of an actual kumite match with an accuracy of 0.875.

## 1. Introduction

Karate is a Japanese martial art that features combat techniques, such as punching, kicking, and defense. There are two main types of karate competitions: kata and kumite. We focus on kumite in this study. Kumite is a karate sparring competition in which two karate players fight each other using various techniques. In kumite, the player can win a match by scoring more points than the opponent by attacking the opponent or defending against the opponent’s attacks. Because most kumite techniques are linear and very fast [1], anticipating techniques during defense is important. In order to predict the opponent’s attack, reading an opponent’s preliminary actions (hereinafter referred to as “pre-action”) during the attack is effective. On the other hand, if the opponent is not aware of the player’s pre-action, the attack is more likely to succeed.

The pre-action is the motion that occurs just before the attack technique in kumite, such as fist movement, arm lowering, and shoulder raising. Petri et al. found that punch techniques were most likely to be recognized by the opponent in the preparation phase of the attack by analyzing the “anticipatory cues” in a kumite match [2]. The anticipatory cues that take place during the attack preparation phase are the pre-actions, which inform the opponent of the timing of the attack and give the opponent time to prevent the attack.

Therefore, accurately grasping and reducing pre-actions is important for a successful attack. However, players experience difficulty in accurately recognizing their pre-actions because the pre-action is performed subconsciously in many cases.

We have proposed a method to estimate the presence or absence of the pre-action based on the similarity between the acceleration data of an arbitrary punch and a previously prepared dataset consisting of the acceleration data of punches without the pre-action [3]. We refer to this method as GLID (gradually lengthening inverted-window dynamic time warping). GLID can only detect the pre-action in a punch performed by a player in a static state, and it is limited to basic practice. Accurately grasping whether a player can punch without pre-action, even during an actual kumite match, is necessary to improve the skills of a kumite match.

The main difference between punching from a static state and punching during a kumite match is whether or not footwork is performed before and after the punching. Here, “footwork” refers to controlling the movement of the feet to adjust position and posture in relation to the opponent. Players basically perform footwork between kumite techniques during kumite matches.

From a signal processing perspective, a major challenge arises when applying GLID to actual matches: the inertial data of a punch often contains both pre-action and footwork components. GLID estimates the presence or absence of pre-action by calculating the DTW (dynamic time warping) distance between the punch data to be analyzed and a reference dataset of punches without pre-action. However, if the analyzed punch data contains sufficient footwork, even punches without pre-action may result in large DTW distances. This can cause the algorithm to misrecognize the inertial data waveform of footwork as that of pre-action. Without properly removing the footwork component, reliably detecting true pre-actions becomes difficult.

Therefore, in this study, we introduce a new method designed to address the complexities associated with actual kumite match scenarios. We propose GWA-GLID, a method for detecting the pre-action in kumite matches by combining GLID with preprocessing the inertial data during kumite matches to make it applicable to GLID. The preprocessing, named GWA (gradually window-shrinking autocorrelation function), can effectively identify and smooth footwork segments. We can reliably distinguish the pre-action from the footwork by using GWA. We conducted an experiment to verify the accuracy of detecting the pre-action during kumite matches using this method. As a result, we found that the proposed method can detect pre-actions in punches during a kumite match with an accuracy of 0.875.

The main contributions of this paper are as follows.

- We proposed GWA, an algorithm that automatically detects footwork segments in inertial sensor data and smooths them to suppress their influence.
- We introduced GWA-GLID, a hybrid method that combines GWA with GLID, enabling the reliable detection of pre-actions during kumite matches.
- We validated the effectiveness of GWA-GLID through experiments on actual kumite match data, demonstrating significant performance gains over the conventional method.

The rest of this paper is organized as follows: Section 2 introduces related research, Section 3 explains the proposed method, Section 4 evaluates the proposed method, and Section 5 summarizes the paper.

## 2. Related Work

In this section, we review related work to clarify the novelty and positioning of this study. First, we introduce studies that evaluate karate movements using various sensor-based methods and highlight the importance of focusing on pre-actions. Next, we introduce studies on motion recognition using inertial sensors and explain our rationale for using inertial sensors and DTW. Finally, we summarize existing approaches for the automatic segmentation of time-series data.

### 2.1. Evaluation of Karate Movements Using Sensors

Many studies have used various sensors to evaluate karate motions and assist in practice. Motion capture was used to classify karate kicks [4,5] and analyze kata movements [6]. Although motion capture systems can visualize and evaluate detailed karate motions, it has been pointed out that the use of many markers attached to the body restricts motion. They are also expensive, require large equipment, and need ample space, making them difficult to apply in general use.

In addition, studies analyzing karate experts’ movements using EMG (electromyography) investigated the muscles that are strongly activated when punching [7], the correlation between the punch force and biomechanical parameters [8], and the effect of three months of training on the electromyographic activity during punching and kicking [9]. There are studies using computer vision to develop the karate smart coaching system [10], to evaluate karate performance [11], and to classify kumite movements [12]. Computer vision techniques may be unable to analyze micro movements during kumite, depending on the camera angle.

In this way, there are many studies related to karate that utilize sensors. However, most focus on classifying kumite techniques or evaluating their accuracy. No studies addressed the pre-action, one of the important elements in kumite matches. Therefore, we analyze the pre-action in karate using inertial sensors, which have recently gained widespread use in sports and martial arts research.

### 2.2. Motion Recognition Method Using the Inertial Sensor

Many methods have been studied to recognize actions such as posture, motion, and gestures using accelerometers, gyroscopes, and IMUs (inertial measurement units). Zmitri et al. proposed the subspace KNN (k-nearest neighbor) classifier that recognizes seven activities or postures using three IMUs [13]. Mao et al. proposed a hybrid HAR (human action recognition) approach that achieved a high accuracy of 0.96 across 19 different human pose recognition tasks using an MLP (multi-layer perceptron) neural network and Euler angle extraction based on IMUs [14]. Pernek et al. developed a system with five IMUs to recognize the type and intensity of arm muscle training with a barbell using a two-layered SVM (support vector machine) [15]. In addition, other studies classified human posture using RF (random forest) [16] or LSTM (long short-term memory) [17] and compared the accuracy of multiple classifiers [18]. Junker et al. [19] and Georgi et al. [20] proposed a method to recognize specific gestures using HMM (hidden Markov model) and achieved high recognition accuracy.

In a study of karate motions using inertial sensors, Vuković et al. analyzed in detail the punches of advanced players using two inertial sensors to significantly improve their proficiency in kumite techniques [21]. Yadav et al. proposed MS-KARD, a multi-stream karate action recognition dataset using two cameras and three inertial sensors [22]. Labintsev et al. proposed a model that could classify five types of punches with an accuracy of 0.96 using an IMU attached to the fist [23].

In this way, motion analysis using inertial sensors is known for its high accuracy, and its analysis methods are varied. In this study, we focused on DTW as an analysis method for inertial data. DTW is an algorithm that can measure the similarity of time series data even if they have different lengths and periods. DTW measures the similarity between two time series by exhaustively calculating the distance between each pair of points. Many studies have modified DTW to meet specific research objectives, such as the two-dimensional dynamic time warping algorithm (2D-DTW) [24], comparative dynamic time warping (C-DTW) [25], and SegrDTW [26]. We also proposed GLID to estimate the presence or absence of the pre-action by calculating the DTW distance between the measured acceleration data and a prepared dataset without pre-action [3]. The karate techniques and pre-actions that are the objects of recognition in this study do not have fixed durations or consistent speeds. Therefore, we believe DTW is suitable for their recognition.

### 2.3. Time Series Data Analysis with Automatic Segmentation

Accurately dividing the footwork segment before the punch, the pre-action segment, and the punch action segment is necessary to detect the pre-action in a punch during a kumite match. Much research has been conducted on automatic segmenting time series data to understand motion patterns and detect change points where gestures switch. Darkhovsky et al. introduced the concept of the complexity of a continuous function defined on an interval and detected and segmented change points in arbitrary nature time series data by characterizing classes of Hölder continuous functions [27]. Inoue et al. proposed unsupervised layered segmentation that can be used as a pre-processing step for annotating time series data consisting of several types of gestures [28]. Moreover, Vögele et al. proposed an efficient method for the fully automatic temporal segmentation of human behavior [29].

In addition, because motion such as “walking” and “running” is a repetition of certain data, there is research on the segmentation of time-series data by calculating the value of the ACF (auto-correlation function) of the acceleration data. Yang et al. proposed a wearable acceleration measurement system for real-time gait cycle parameter recognition using an autocorrelation procedure [30]. They quantified and distinguished gait cycle parameters between two subject groups of different mobility abilities by measuring trunk acceleration during walking. Jain et al. recognized stride boundaries using the ACF for automatic stride segmentation in different walking activities [31].

It has been shown that the ACF can be effectively segmented for constant time-series data. The footwork during a kumite match is also constancy motion. Therefore, we propose a method of separating the footwork segment and the kumite technique segment by using the ACF to determine the constancy of the acceleration data during the kumite match.

Previous studies have shown the effectiveness of automatic segmentation techniques in identifying repetitive or consistent motion patterns. However, these techniques have not been applied to the detection of footwork in martial arts or its separation from pre-actions. In this study, we address the limitations of conventional methods by introducing autocorrelation-based segmentation optimized for the motion patterns characteristic of kumite.

## 3. Proposed Method: GWA-GLID

While various sensor-based and machine learning approaches have been applied to karate motion analysis, the existing methods face significant challenges in accurately detecting pre-actions during kumite matches. To address these limitations, we propose GWA-GLID, a novel method that estimates the presence or absence of pre-actions during kumite matches by combining preprocessing based on constancy decisions with GLID. This approach enables more robust and precise identification of pre-actions in actual match scenarios.

**Figure 1:**
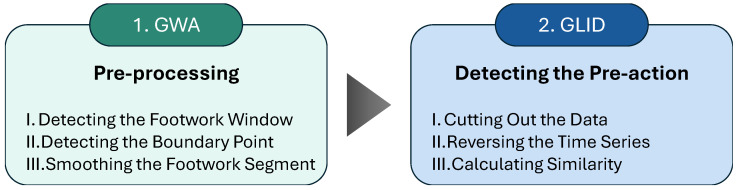
Flow of GWA-GLID.

A flow of GWA-GLID is shown in Figure 1.

GWA is a pre-processing for detecting and smoothing footwork segments in inertial data during a kumite match. GLID is a method for estimating the presence or absence of the pre-action in a static state, which we previously proposed [3]. In GWA-GLID, GWA is first performed as a preprocessing step, followed by GLID to estimate the presence or absence of pre-actions. In the following, we first describe GLID as the conventional method of pre-action detection, followed by GWA as the pre-processing method. Here, in GWA-GLID, we applied GWA to three axes of the acceleration data. Based on the results, we applied GLID to six axes data consisting of three axes of acceleration and angular velocity data.

### 3.1. GLID: Gradually Lengthening Inverted-Window Dynamic Time Warping

In this section, we summarize the content of our previous paper [3] on GLID, a method for estimating the presence or absence of pre-actions to facilitate the understanding of GWA-GLID.

#### 3.1.1. Overview of GLID

**Figure 2:**
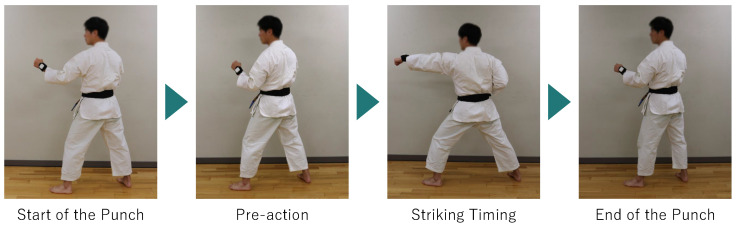
How to perform the forefist punch.

GLID is a method designed to estimate the presence or absence of pre-actions in forefist punches performed in a static state by leveraging DTW. In this study, we focus on the forefist punch, the most basic kumite technique, explained in Figure 2. Figure 2 shows, as an example, the flow when a player has performed a pre-action before the forefist punch. What is important here is that pre-actions are not performed for every punch. The size of the pre-action also varies, and when the pre-action is small, it becomes harder for the opponent to detect the attack. Figure 2 shows an example where a large pre-action is performed.

Forefist punches are most commonly used in kumite matches by karate experts [32]. Moreover, Petri et al. showed that the forefist punch is more often predicted by pre-actions than other kumite techniques in kumite matches [33]. Additionally, we define “striking timing” as when the arm is fully extended and the fist reaches the target point.

Detecting the pre-action in the forefist punch using conventional gesture recognition methods based on inertial sensors is difficult due to the following three features of the pre-action. First, the waveform of the pre-action acceleration data is smaller than those of other gestures such as punch and kick. This feature makes it difficult to detect only the pre-action part in a series of gestures. Next, the pre-action is included during the gesture; therefore, detecting the pre-action is difficult with methods that identify the transition points in continuous motion where the gesture switches such as automatic segmentation or detection of motion occurrence timing. Finally, constructing a dataset labeled for each type of pre-action is difficult because the waveform of the pre-action inertial data in the forefist punch is indefinite. Therefore, we proposed GLID, a method for estimating the presence or absence of the pre-action in the forefist punch by taking these features into account.

#### 3.1.2. Dataset Without Pre-Action

As a preliminary preparation for estimating the presence or absence of the pre-action in an arbitrary forefist punch (hereinafter referred to as “input data”), we prepared a dataset without pre-action consisting only of inertial data from the forefist punch without pre-action (hereinafter referred to as “dataset without pre-action”). We created the dataset without pre-action by collecting the inertial data of 10 forefist punches performed by five karate experts belonging to Kobe University’s karate club. In our previous study [3], we constructed the dataset without pre-action, only with three axes of acceleration. In the present study, we extend the dataset to six axes of inertial data by adding the three axes of angular velocity.

**Figure 3:**
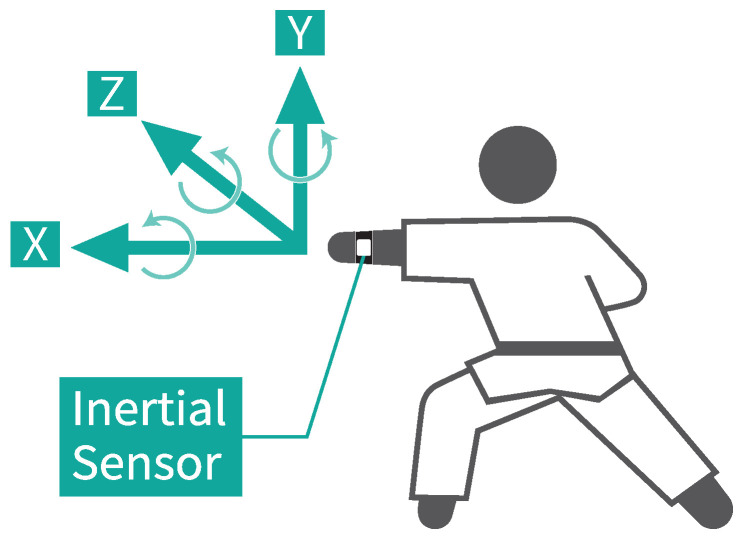
Mounting position of the inertial sensor and the direction of the six axes.

**Figure 4:**
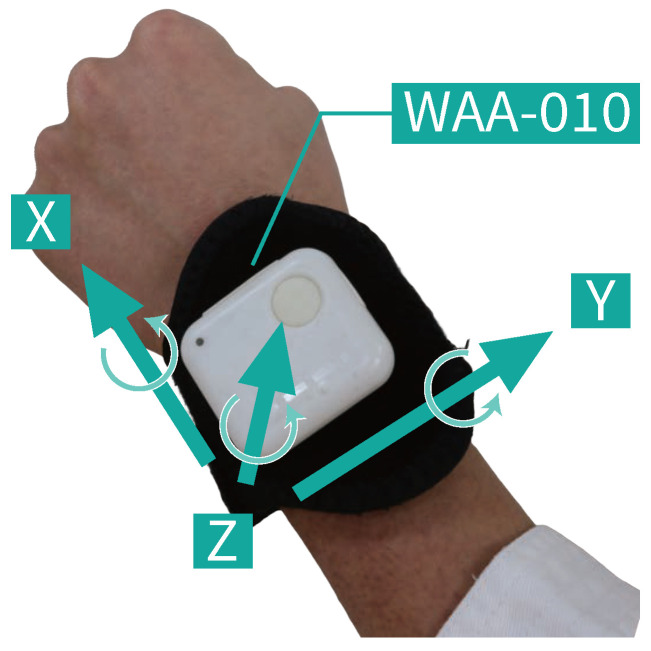
WAA-010, which is the inertial sensor used in this study, and its axial direction.

We measured the inertial data with an inertial sensor attached to a stretchable wristband on the wrist of the side performing the forefist punch, as shown in Figure 3. We used a compact wireless hybrid sensor II (WAA-010) from ATR-Promotions (Kyoto, Japan) for the measurements, as shown in Figure 4. The directions of the three axes, X, Y, and Z, of the accelerometer and gyroscope, respectively, for a total of six axes, are shown in Figure 3 and Figure 4. We used a ThinkPad X13 Gen 2 PC (OS: Windows 10 Home, CPU: 11th Gen Intel® Core^TM^ i5-1135G7 @ 2.40 GHz, RAM: 16.0 GB) from Lenovo (Beijing, China). We obtained the inertial data using the AccelViewerHybrid-II (ver.2.4.0) WAA-010 dedicated data receiving software.

#### 3.1.3. Estimation Method for the Presence or Absence of the Pre-Action

**Figure 5:**
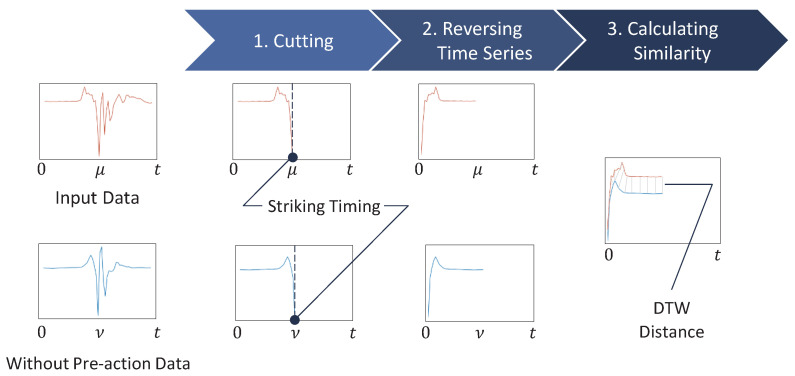
Overview of GLID.

GLID is a method for estimating the presence or absence of the pre-action based on the DTW distance calculation results of the dataset without pre-action and the input data. An overview of GLID is shown in Figure 5.

Let μ and ν be the index of the striking timing in time series data of input data and data without pre-action in Figure 5. The gray lines in Step 3 in Figure 5 are the lines connecting the points with the smallest DTW distance at each point of the two time series data.

First, we cut out the data from the start of the forefist punch to the striking timing in order to analyze the inertial data, focusing only on the pre-action part. We identify the striking timing by detecting the negative peak in the acceleration data on the X-axis, which occurs just before the punch reaches its maximum velocity.

Next, we reverse the time series of the cutout input data and the dataset without pre-action and calculate their DTW distance. When and how the pre-action is performed varies, and its acceleration and angular velocity waveforms differ each time. Therefore, accurately detecting the presence or absence of a pre-action before a forefist punch is difficult, even if the measurement of DTW distance is started at an appropriate point in the time series. Here, the inertial waveform just before the negative peak of a punch is similar, regardless of whether the pre-action is present. We can focus only on the pre-action waveforms that precede similar waveforms while maintaining the consistency of the number of peaks in the pre-action waveforms by setting the striking timing of the forefist punch to the uniform starting point of the DTW and reversing the time series. We can estimate the presence or absence of the pre-action based on whether a corresponding pre-action waveform exists following this similar waveform.

**Figure 6:**
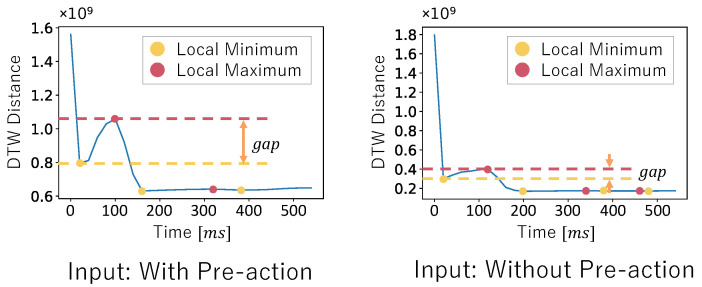
Comparison of DTW distance trend graphs in the presence and absence of the pre-action.

Finally, we involve incrementally extending the time window of the input data and calculating the DTW distance to the dataset without pre-action sequentially. We calculated the DTW distance using the method of Myers et al. [34]. By tracking how this distance evolves as the input data includes more of the pre-action phase, we observe that the presence of the pre-action typically causes a noticeable increase in the DTW distance. This is because the waveform patterns during the pre-action differ significantly from those without the pre-action. We analyze the DTW distance graphs by identifying first the local maximum and minimum, as shown in Figure 6. The gap between these points serves as a reliable indicator of the pre-action in the input data.

To quantify this, we define a threshold based on the result of the preliminary investigation, which allows us to classify whether the input data contains the pre-action. If the gap between the local maximum and minimum exceeds this threshold, we estimate that the pre-action is present; otherwise, it is absent.

Here, in Figure 6, the initial DTW distance is very large because the time window of the input data is initially very small. At this stage, regardless of the data content, the waveform significantly differs from the dataset without pre-action. The DTW distance then becomes smaller as the time window of the input data is increased.

Additionally, without reversing the time series, we start DTW calculations from arbitrary points, causing waveform misalignment and reducing detection accuracy. The pre-action phase waveforms do not align, making it difficult to consistently calculate the gap between the local maximum and minimum, which we use to detect pre-actions. Therefore, we find that reversing the time series is essential to correctly compare the presence or absence of pre-actions based on waveform similarity.

### 3.2. GWA: Gradually Window-Shrinking Autocorrelation-Function

#### 3.2.1. Overview of GWA

GLID is limited to detecting the pre-action only in basic practice scenarios and could not confirm whether the forefist punch was executed without the pre-action during actual kumite matches. To address this limitation, we propose GWA-GLID, a new method capable of detecting the pre-action even when the player is in motion, as in a kumite match. GWA-GLID is a method that combines GWA, a pre-processing method for detecting and smoothing footwork performed during a kumite match, before GLID.

**Figure 7:**
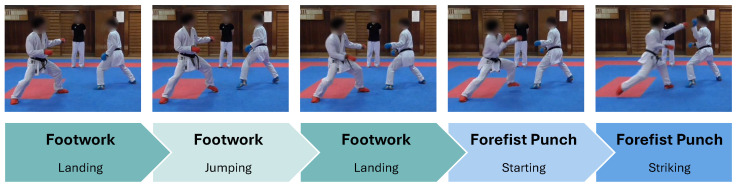
Flow from the footwork to the forefist punch.

One of the key characteristics of kumite matches is that players perform footwork between attacks and defenses. This footwork, which involves hopping in place or moving forward, backward, or sideways using the toes, allows for smoother attacks and makes it difficult for opponents to predict the target or timing of an attack. Figure 7 shows a player wearing red protective gear performing the footwork while repeating jumping and landing several times before the forefist punch.

We are concerned that GLID might misidentify this footwork as the pre-action. Therefore, we consider that recognizing when footwork occurs during a kumite match and smoothing the footwork segment can allow us to estimate the presence or absence of the pre-action in forefist punches performed during the match. In this paper, let the footwork and non-footwork windows denote whether the inertial data in the sliding window occurs during footwork, respectively. In addition, let the footwork and non-footwork segments denote whether the inertial data segment in the kumite match occurs during footwork, respectively.

**Figure 8:**
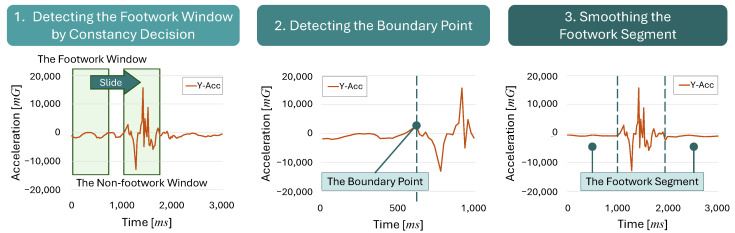
Overview of GWA.

An overview of GWA is shown in Figure 8.

We describe each flow step in detail in the following sections.

#### 3.2.2. Detecting the Footwork Window by Constancy Decision

We classify footwork and non-footwork windows by constancy decisions based on the calculation of the ACF values in the sliding window. We denote the ACF at lag *k* of the time series data x(t), as follows:

(1) $$\begin{array}{c} r_{k}=\frac{\sum_{t=1}^{N-k}\left(x_{t}-\bar{x}\right)\left(x_{t+k}-\bar{x}\right)}{\sum_{t=1}^{N}{\left(x_{t}-\bar{x}\right)}^{2}} \end{array}$$

Let $r_{k}$ be the ACF at lag *k*; $x_{t}$ is the value of the time series data at time *t*, $\bar{x}$ is the mean of the time series data, and *N* is the total number of the time series data.

We calculate the value of ACF using only the acceleration data $D^{\mathrm{Acc}}$ in the inertial data of the forefist punch during a kumite match. This is because we cannot detect the constancy characteristics of the footwork in the angular velocity data. We use Algorithm 1 to determine the footwork window based on the ACF calculation results within the sliding window.
**Algorithm 1** Constancy decision by calculating the value of ACF**Require:** Sliding window data
d(t) for arbitrary axes in $D^{\mathrm{Acc}}$**Ensure:** 
$r_{k}$ and Threshold    **Let** ACF(x(t)) be a method that calculate $r_{k}$ of the acceleration data x(t)    **for** 
k=1,2,…,N 
**do**         $r_{k}=\mathrm{ACF}\left(d\left(t\right)\right)$         **if**
$r_{k}>r_{k-1}\;\mathrm{and}\;r_{k}>r_{k+1}$ **then**             Threshold=α·(1−k/N)             **Break**         **end if**      **end for**

Let *N* be the window size for calculating the value of ACF, which is set to 50 samples (1000 ms) in this paper. We set Threshold in Algorithm 1 based on the method of Murao et al. [35]. Using this algorithm, we can obtain the results of the ACF calculation and Threshold for the constancy decision. Here, we slide the window by five samples (100 ms), overlapping the previous window in this paper.

**Figure 9:**
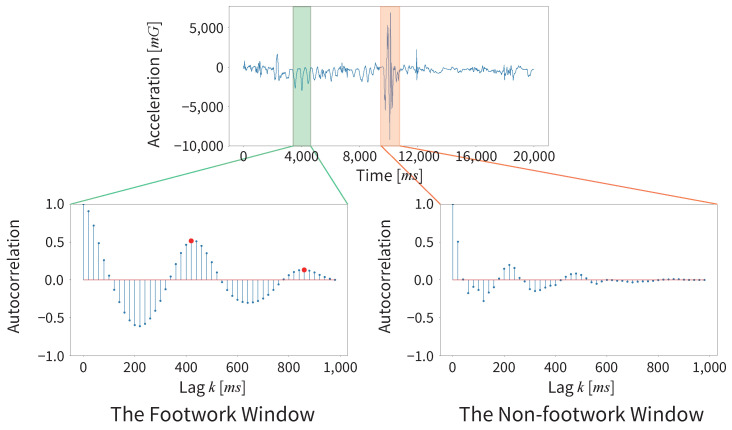
Comparison of the ACF between the footwork window (green window) and the non-footwork window (orange window).

The acceleration waveform of the Y-axis in forefist punching during a kumite match and the ACF for each footwork movement and the kumite technique are shown in Figure 9. As shown in Figure 9, the ACF of the footwork with constancy has definitive peaks.

However, the ACF of the kumite technique without constancy does not show a high peak. In the GWA, if the value of the first peak $r_{k}$ exceeds Threshold, it is determined to be a footwork window.

(2) $$\begin{array}{c} \begin{array}{ccc} \mathrm{if}\;r_{k}\geq Threshold & \Rightarrow & Footwork\;window\\ \mathrm{otherwise} & \Rightarrow & Non\text{-}footwork\;window \end{array} \end{array}$$

The following is an explanation of how to obtain Threshold. The value of ACF decreases linearly with increasing lag *k*, and the peak height of a periodic waveform, such as a sine wave, is 1−k/N. α is the coefficient. We need to set it between 0.1 and 1.0 to obtain the highest accuracy of the GWA for each data to be analyzed.

#### 3.2.3. Detecting the Boundary Point Between the Footwork Segment and the Non-Footwork Segment

We recognize the accurate boundary point between the footwork and non-footwork segments in the following procedure.

First, we estimate which windows are footwork windows for the acceleration along the X, Y, and Z axes using Algorithm 1. The following steps are performed for each axis.

Next, if a certain number of consecutive non-footwork windows appear, we regard the first of these windows as the start of the non-footwork segment (kumite technique segment). We tested the consecutive number of non-footwork windows from 2 to 10 and found that the most accurate result was achieved with 7 consecutive windows.

Then, we detect the accurate boundary point between the footwork and the kumite technique in the window identified as the start of the non-footwork segment, as shown in Algorithm 2.
**Algorithm 2** Detection of the accurate boundary point**Require:** The first detected non-footwork window data $d^{\prime }\left(t\right)$**Ensure:** The boundary point $t_{b}$     **Let** *L* be the number of the time series data $d^{\prime }\left(t\right)$     **Let** $\underset{1\to l}{\mathrm{shrink}}\left(x\left(t\right)\right)$ be a method that shrinks the length of the time series data x(t) from 1 to *l*     **Let** ACFList be a list of the ACF results     **for** 
l=1,2,…,L 
**do**         $s\left(t\right)=\underset{1\to l}{\mathrm{shrink}}\left(d^{\prime }\left(t\right)\right)$         **for** k=1,2,…,N **do**             $r_{k}=\mathrm{ACF}\left(s\left(t\right)\right)$             **if** $r_{k}>r_{k-1}\;\mathrm{and}\;r_{k}>r_{k+1}$ **then**                  Add $r_{k}$ to ACFList                  **Break**             **end if**         **end for**      **end for**      $t_{b}=\mathrm{the}\;\mathrm{index}\;\mathrm{of}\;\max\left(ACF\;List\right)$

**Figure 10:**
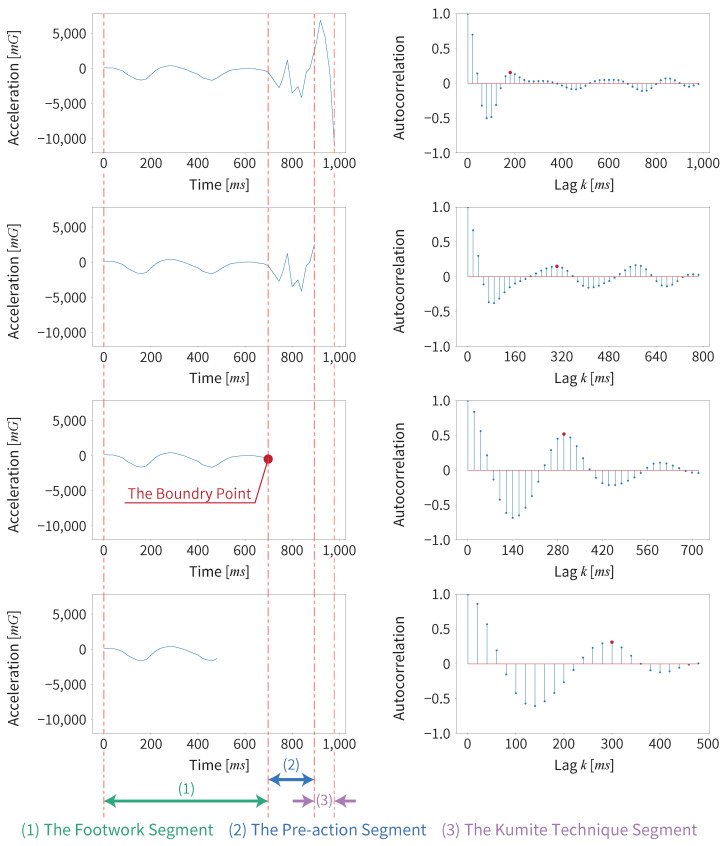
Detection of the boundary point by gradually shrinking window and calculating the value of ACF.

We consider that the first detected non-footwork window consists of “footwork”, “pre-action”, and “kumite technique” in that order if it contains the pre-action. We gradually shrink the length of this window from its end in the time series, and we calculate the value of ACF at each step, as shown in Figure 10.

As the window shrinks, the pre-action and kumite technique parts are gradually excluded. Accordingly, only the constancy footwork part remains, and the value of ACF gradually increases. Specifically, the first positive peak value of ACF is gradually closer to 1. When only the footwork segment remains, the value of ACF is at maximum. If we shorten the window further, the value of ACF begins to become smaller. We consider that the time at which this peak is detected is the boundary point between the constancy segment and the non-constancy segment. We set this boundary point as the start of the non-footwork segment.

We need to use the above process because the length of time at which the footwork constancy becomes detectable differs, depending on the player. If the window size is reduced from the beginning, footwork constancy may not be detected accurately. Therefore, we need to calculate the ACF by gradually shrinking the window size after sliding with a larger window size.

Finally, we apply the same method to detect the end boundary point of the non-footwork segment. If two consecutive footwork windows appear after the start point of the non-footwork segment is detected, we regard the first of these windows as the end of the non-footwork segment. We gradually shrink the length of this window from its start in the time series, and calculate the value of ACF at each step. In this way, we can obtain the end point of the non-footwork segment in the same way as the start point.

#### 3.2.4. Determination of the Overall Boundary Point Between Six Axes

We determine the overall boundary point based on each result of the boundary points of the footwork and non-footwork segments detected for the three axes of the acceleration data. The overall boundary points are obtained through logical operations applied to the footwork segments detected for each axis.

Let $S_{X},S_{Y}$ and $S_{Z}$ be the detected non-footwork segments for the X, Y, and Z axes acceleration, respectively.

First, we apply a logical conjunction (AND operation) to the X and Z axes results, detecting motion in both horizontal directions.

(3) $$\begin{array}{c} S_{XZ}=S_{X}\wedge S_{Z} \end{array}$$

Here, the logical conjunction (∧) extracts the overlapping segments in the footwork segments of both axes. The footwork rarely involves only back-and-forward or side-to-side motion. Therefore, we need to detect the boundary points by placing more emphasis on the results of the Y-axis, which represents up-and-down motion.

Next, we take the logical sum (OR operation) of $S_{XZ}$ with the Y-axis result.

(4) $$\begin{array}{c} S_{\mathrm{final}}=S_{XZ}\vee S_{Y} \end{array}$$

Here, the logical sum (∨) combines the footwork segments detected in each axis, ensuring that any motion detected in at least one axis contributes to the final boundary determination.

However, in cases where this strict condition fails to detect the boundary points, we apply a more relaxed approach. In this case, we apply a logical sum across all three axes.

(5) $$\begin{array}{c} S_{\mathrm{final}}=S_{X}\vee S_{Y}\vee S_{Z} \end{array}$$

This approach prevents missed detections by including any detected footwork along any of the three axes.

Using these logical operations, we determine the boundary points between the footwork and non-footwork segments for forefist punches during kumite matches. We apply the boundary points determined by the above approach to all six axes, for a total of three axes of acceleration and three axes of angular velocity.

#### 3.2.5. Smoothing the Footwork Segment by Moving Average Method

We apply the moving average method to only the footwork segment determined in Section 3.2.4. We perform the process on all six axes of data, including the three axes of angular velocity data. The window size for calculating the moving average is set to 50 samples (1000 ms). This smoothing process prevents the inertial data of footwork and pre-action from being recognized as similar waveforms when calculating the DTW distance by adapting GLID to the inertial data of forefist punches during a kumite match.

## 4. Evaluation Experiment

We conducted an experiment to verify the accuracy of GWA-GLID for estimating the presence or absence of the pre-action in the forefist punch during a kumite match.

### 4.1. Experimental Setup

**Figure 11:**
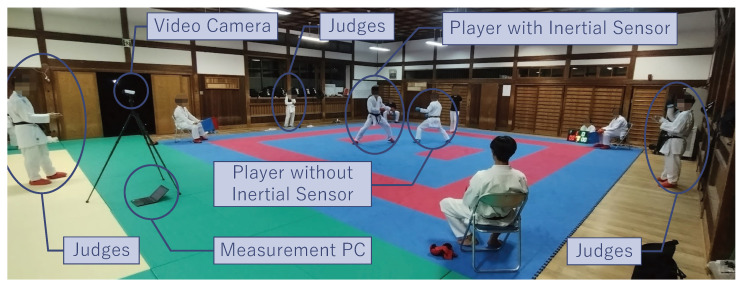
The experimental environment.

**Figure 12:**
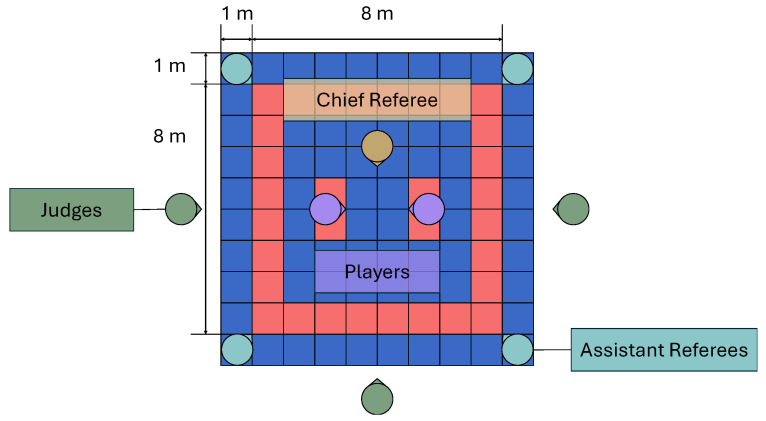
The size of the court and the experimental arrangement.

We conducted the experiment in an environment, as shown in Figure 11 and Figure 12.

The participants were four karate players (two teenage males and two males in their 20s) who belonged to a university karate club and had been practicing karate for under three years. Moreover, we asked three karate players with over three years of experience from a university karate club (hereafter referred to as “judge”) to evaluate the presence or absence of pre-actions. The judges were positioned outside the court and visually judged the presence or absence of pre-actions in the forefist punches executed by the participant wearing the inertial sensor. The judges recorded on the evaluation sheets, in sequential order, whether each forefist punch performed during the match included a pre-action. Additionally, we had one chief referee standing inside the court, while four assistant referees were seated at the corners of the court, similar to an actual kumite match. The matting material of the coats used in the experiment was polyolefin foam.

In this experiment, the participants conducted kumite matches with each other. During each match, we equipped only one of the participants with an inertial sensor and recorded the acceleration and angular velocity data throughout the match.

We measured data by attaching the compact wireless hybrid sensor II (WAA-010) from ATR-Promotions (Kyoto, Japan) used in Section 3.1.2 to the wrist of the participant’s punching hand. If we attach the inertial sensor to the participant with the X-axis acceleration in the wrong direction, we will not be able to detect the striking timing of the forefist punch, and the GLID process will not proceed. In addition, if we misorient the Y-axis acceleration, the accuracy of the boundary point between the footwork and the non-footwork segment will be reduced. Therefore, we need to mount the inertial sensor in the correct orientation.

We set the duration of the matches to two minutes and collected data for eight matches. The participant executed with the inertial sensor performed 51 forefist punches during eight matches. We assigned the ground truth label for the presence or absence of pre-actions for each forefist punch after the matches. By reviewing the evaluation sheets collected from the judges, we labeled each forefist punch as “with pre-action” if two or more of the judges evaluated that the pre-action was included; otherwise, the label was “without pre-action”.

In this experiment, we manually extracted the sections of the inertial data where forefist punches were performed. First, we detected the negative peak values in the overall acceleration data of the kumite matches. We considered these points to indicate the timing of some kumite techniques, including forefist punches. Next, we identified the specific times when forefist punches occurred among the detected negative peak points by visually referencing the recorded video of the kumite matches and the graphs of the inertial data. The kumite matches were recorded using a video camera placed outside the court. Finally, we extracted segments of 300 samples (6 s) before and after these points. This duration was sufficient to capture the constancy of the footwork.

Furthermore, we set the coefficient α described in Section 3.2.2 to 0.5. This value was determined by adjusting α from 0.1 to 1.0 in increments of 0.1.

This experiment was conducted with the approval of the Ethical Review Committee for Research Directly Involving Human Participants of the Graduate School of Engineering, Kobe University (approval numbers 05–11).

### 4.2. Result

The result of the comparison of the detection accuracy for the pre-action in forefist punches during actual kumite matches using the proposed method (GWA-GLID) and the conventional method (only GLID) is shown in Table 1. The accuracy was higher when the six axes of data were used compared to when only the three axes of acceleration data were used for both methods.

We conducted a *t*-test for the detection accuracy of the pre-action between the proposed method and the conventional method for each participant’s forefist punches during the kumite matches. The results showed that the proposed method had significantly higher accuracy (*t*(3) = 4.23, *p* < 0.05). The precision was 0.789, indicating that the GWA-GLID incorrectly estimated the presence of the pre-action in four forefist punches with no pre-action. On the other hand, the recall reached 1.00, indicating that all forefist punches with the pre-action were correctly identified as having the pre-action. The F-score was 0.882, indicating a good balance between precision and recall. The result of the F-score shows that while the GWA-GLID effectively detects pre-actions during a kumite match, there is room for improvement in reducing misclassifications.

### 4.3. Discussion

The results presented in Section 4.2 demonstrate the effectiveness of the proposed method for detecting pre-actions during kumite matches. This is evidenced by improvements in accuracy, precision, and recall compared to the conventional method. In this section, we interpret these results in detail and discuss issues that need to be resolved for future practical applications.

First, a limitation of this study is the relatively small sample size, both in terms of the number of participants and the total number of forefist punches analyzed. A larger and more diverse dataset is necessary to validate the generalizability and robustness of the proposed method.

**Figure 13:**
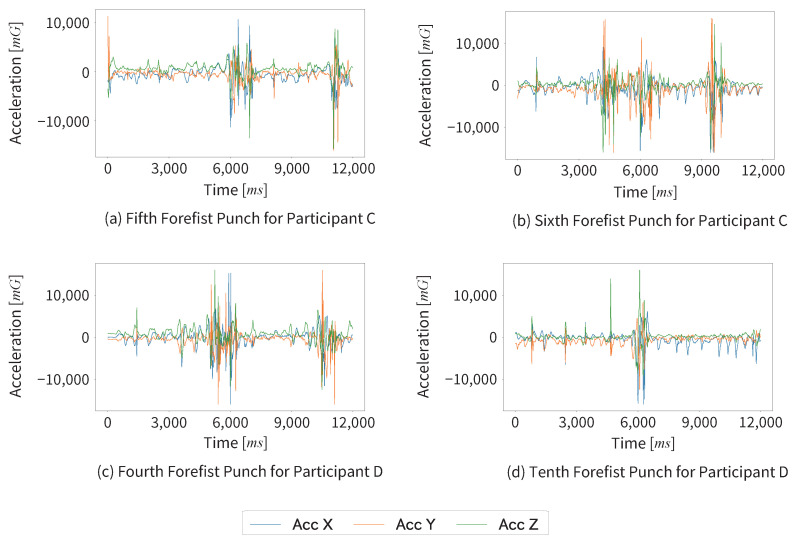
The three axes acceleration data graphs of the four failed segmentation cases.

**Figure 14:**
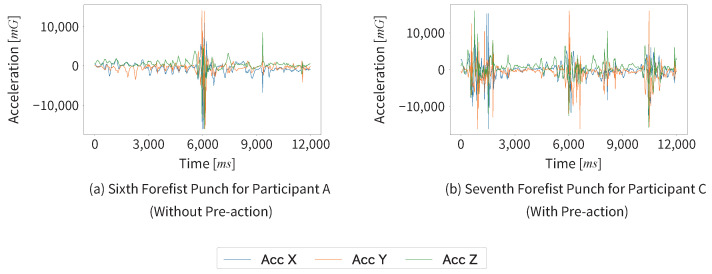
The three axes acceleration data graphs of the two successful segmentation cases.

Next, we could not segment the footwork and non-footwork segments in four data among 51 data of forefist punches recorded during the kumite matches in this experiment. The three axes acceleration data graphs of the four failed segmentation cases and two successful segmentation cases are shown in Figure 13 and Figure 14.

We found that Participants A and B successfully segmented all data; in contrast, Participants C and D each experienced two segmentation failures. Participants C and D had one year less training experience than Participants A and B, and there were differences in the quality of their forefist punches. This could be one of the contributing factors to the segmentation failures. In the failed cases (a), (b), and (c), constancy may have been mistakenly detected because the non-footwork segments were longer than the successful cases. In the failed case (d), although the cause is difficult to determine, similar acceleration waveforms appeared to repeat in the X and Y axes during the forefist punch.

Finally, adjusting the coefficient α, used as Threshold for detecting footwork segments, proved difficult. We set α to 0.5, as it was the most effective value for the footwork data of the four participants. However, we might need to adjust α when the footwork being analyzed changes. Lowering α makes it easier to recognize the data within the sliding window as footwork, improving the estimation accuracy of the boundary points between the footwork and non-footwork segments. However, if α is set too low, the sliding windows containing the forefist punch may also be recognized as footwork windows, resulting in failure to detect the boundary points. We consider that this was likely the cause of the second limitation. By contrast, increasing α makes it harder to identify footwork within the sliding window, reducing missed detections of forefist punches. However, the estimation accuracy of the boundary points is lower. Thus, finding the appropriate setting for α remains a challenge.

Furthermore, considering the potential impact of pre-action detection errors on training and player performance is important. If the proposed method incorrectly detects a pre-action when none is actually present, there is a risk that players may unnecessarily change the correct punching form, thereby impeding skill development. Conversely, if the method fails to detect an existing pre-action, players may continue to practice and internalize incorrect techniques without realizing their mistakes. Therefore, improving the accuracy of the proposed method is a high priority, and addressing the aforementioned challenges is essential to ensure its effectiveness and reliability.

Compared to previous related work, our method offers the advantage of accurately distinguishing subtle pre-actions from repetitive footwork during kumite matches, a challenge that most existing approaches have not addressed. However, unlike some deep learning-based approaches that can leverage large datasets for general action recognition, the performance of our method still depends on careful parameter tuning. This limitation remains an area for future improvement.

## 5. Conclusions

In this paper, we proposed GWA-GLID, a method for detecting pre-actions in forefist punches performed during actual karate kumite matches. The detection accuracy of the pre-action is low with the conventional method that is limited to a static state because players are performing constancy footwork during a kumite match. Therefore, we proposed GWA, a method that calculates the value of ACF using a sliding window to detect and smooth only the footwork segment within the inertial data of a kumite match. We conducted experiments to evaluate the effectiveness of applying GWA to improve the detection accuracy of the pre-action in forefist punches during kumite matches. The results showed that applying GWA improved the accuracy by 0.208 over the conventional method, achieving an accuracy rate of 0.875 in detecting pre-actions.

In the future, we first plan to increase the number of participants and collect samples and conduct additional experiments to further evaluate the effectiveness of GWA-GLID.

Then, we need to develop an automatic optimization method for Threshold coefficient α. This approach aims to eliminate cases where the boundary points between the footwork and non-footwork segments cannot be detected.

Additionally, we need to develop a method that can automatically recognize the segments during kumite matches, types of kumite techniques, and the presence or absence of pre-actions. We believe that this approach can be achieved in the following steps. First, we will construct the dataset without the pre-action of not only the forefist punch but also each kumite technique. Next, we will develop a machine learning model capable of classifying the types of kumite techniques using these datasets. Then, we will analyze the data segments detected around the acceleration peaks during the kumite match using the developed machine learning model to classify the types of techniques. Finally, we will apply the proposed method from this study to estimate the presence or absence of pre-actions.

## Figures and Tables

**Table 1:** Comparison of detection accuracy of pre-actions in forefist punches during actual kumite matches.

	GLID	GWA-GLID
Accuracy	0.667	0.875
Precision	0.667	0.789
Recall	0.737	1.000
F-measure	0.700	0.882

## Data Availability

Data were not published due to ethical restrictions. If you want to browse the data, please contact the corresponding author personally.
